# 奥沙利铂或顺铂联合足叶乙甙治疗老年广泛期小细胞肺癌的随机对照临床研究

**DOI:** 10.3779/j.issn.1009-3419.2013.01.04

**Published:** 2013-01-20

**Authors:** 丹 蒲, 梅 侯, 之曦 李, 晓梅 曾

**Affiliations:** 610041 成都，四川大学华西医院肿瘤中心 Cancer Center, West China Hospital, Sichuan University, Chengdu 610041, China

**Keywords:** 足叶乙甙, 奥沙利铂, 顺铂, 小细胞肺癌, 老年, Etoposide, Oxaliplatin, Cisplatin, Small cell lung cancer, The elderly

## Abstract

**背景与目的:**

足叶乙甙（VP-16）联合顺铂（DDP）是广泛期小细胞肺癌（small cell lung cancer, SCLC）一线联合化疗中最常用的方案，但顺铂的恶心呕吐毒副反应影响患者的生存质量。本研究拟比较足叶乙甙联合奥沙利铂或顺铂一线治疗老年广泛期SCLC的疗效及毒副反应。

**方法:**

未经抗肿瘤治疗的老年广泛期SCLC患者71例，随机分成两组，EO组（足叶乙甙80 mg/m^2^第1-5天+奥沙利铂130 mg/m^2^第1天静脉滴注，每21天重复）35例，EP组（足叶乙甙80 mg/m^2^第1-5天+顺铂25 mg/m^2^第1-3天静脉滴注，每21天重复）36例，至少治疗2周期以后评价疗效及不良反应。

**结果:**

EO组与EP组相比，治疗缓解率（55.9% *vs* 54.3%, *P*=0.894），疾病控制率（82.4% *vs* 77.1%, *P*=0.591），中位无进展生存期（5.5个月*vs* 4.7个月，*P*=0.638），中位生存时间（10.5个月*vs* 9.1个月，*P*=0.862）差异均无统计学意义; 毒副反应方面，EO组恶心呕吐等消化道反应发生率低于EP组（65.7% *vs* 97.2%, *P*=0.001），但Ⅰ级-Ⅱ级神经毒性发生率高于EP组（74.3% *vs* 11.1%, *P* < 0.001）。

**结论:**

足叶乙甙联合奥沙利铂或顺铂两种方案用于一线治疗老年广泛期SCLC的疗效相当，但EO组患者耐受性相对较好。

## Abstract (en)

**Background and objective:**

Etoposide combined cisplatin (EP) is the most commonly-used first-line treatment combination chemotherapy regimen in the treatment of extensive-stage small cell lung cancer (SCLC), The side-effects of cisplatin, such as nausea and vomiting, influence patients' quality of life. This study aims to compare the efficacy and toxicities between etoposide plus oxaliplatin (EO) and etoposide plus cisplatin (EP) regimens as first-line chemotherapy for elderly patients with SCLC.

**Methods:**

Seventy-one old, extensive-stage SCLC patients, who had not received anti-tumor treatment, were randomly divided into two groups, namely, EO group (etoposide: 80 mg/m^2^ d1-5+oxaliplatin; 130 mg/m^2^ d1; repeated every 21 days) and EP group (etoposide: 80 mg/m^2^ d1-5+cisplatin; 25 mg/m^2^ d1-3; repeated every 21 days). Efficacy and toxicities were evaluated after 2 or more cycles.

**Results:**

No statistical differences were observed between the EO and EP groups in the response rate (55.9% *vs* 54.3%, *P*=0.894), disease control rate (82.4% *vs* 77.1%, *P*=0.591), median progression free survival (5.5 months *vs* 4.7 months, *P*=0.638), and median survival time (10.5 months *vs* 9.1 months, *P*=0.862). In terms of toxicities, the incidence of nausea/vomiting in the EO group was significantly lower than that in the EP group (65.7% *vs* 97.2%, *P*=0.001), but the neurotoxicity of grade 1-2 in the EO group was more significant (74.3% *vs* 11.1%, *P* < 0.001).

**Conclusion:**

The clinical efficiency of EO and EP regimens is similar to the first-line chemotherapy for extensive-stage SCLC in elderly patients. However, the tolerance of EO regimens is better than that in the EP regiments.

小细胞肺癌（small cell lung cancer, SCLC）的发病率约占肺癌的15%。近些年来，尽管一些发达国家的SCLC发病率正呈现一种下降的趋势^[1]^，我国SCLC的发病率仍处于较高的水平。SCLC易在早期形成远处转移，约2/3的患者在确诊时已进展为广泛期，因此化疗是SCLC患者治疗的主要手段。目前足叶乙甙与铂类联合是广泛期SCLC一线化疗最常用的方案^[2]^。研究^[3]^早已证实其联合应用较依托泊苷单药口服的疗效更好。广泛期SCLC的治疗目的主要在于缓解症状、提高生活质量和延长生存期，目前的治疗手段很难达到长期生存^[4]^。然而老年SCLC患者对顺铂的耐受性差，尤其是恶心呕吐等毒副作用严重影响了患者的生存质量。因此，对于老年SCLC患者的化疗更应重视治疗相关毒副反应。本研究为前瞻性随机临床对照试验，旨在比较足叶乙甙联合奥沙利铂与足叶乙甙联合顺铂一线治疗广泛期老年SCLC的疗效及毒副作用。

## 1 资料与方法

### 1.1 纳入标准与排除标准

纳入标准：2006年3月-2011年3月经组织学或细胞学确诊为广泛期SCLC且年龄≥70岁的老年患者，体能状况评分（performance status, PS）0分-2分，化疗前血常规：白细胞计数≥4×10^9^/L、中性粒细胞绝对值≥2×10^9^/L、血红蛋白≥80 g/L、血小板计数≥100×10^9^/L，肝肾功能和心电图检查均正常，预计生存期 > 3个月。排除标准：PS评分≥3分、出现脑转移、重要脏器功能不全、合并严重的心血管疾病和控制不佳的糖尿病、合并其它恶性肿瘤、曾接受过抗肿瘤治疗。

### 1.2 临床资料

入组71例广泛期SCLC患者，其中男性59例，女性12例，中位年龄73岁。经随机分组，EO组35例，EP组36例。两组患者基线特点如男女比例、中位年龄、PS评分、吸烟等经统计检验无明显差异（表 1）。

**1:** 本研究纳入患者的临床资料（*n*=71） Clinical characteristics of patients included in this study (*n*=71)

Characteristic	EO group (*n*=35)	EP group (*n*=36)	Total	*P*
Gender				0.562
Male	30	29	59	
Female	5	7	12	
Age (year)				0.731
Median	73	72	-	
Range	70-83	70-79	-	
Performance score				0.350
0-1	26	30	56	
2	9	6	15	
Cigarette smoking				0.769
Smoker	31	30	61	
Non-smoker	4	6	10	
EO group: etoposide 80 mg/m^2^ d1-5 and oxaliplatin 130 mg/m^2^ d1 by intravenous infusion, repeated every 21 days; EP group: etoposide 80 mg/m^2^ d1-5 and cisplatin 25 mg/m^2^ d1-3 by intravenous infusion, repeated every 21 days.				

### 1.3 治疗方案

EO组接受足叶乙甙80 mg/m^2^第1-5天，奥沙利铂130 mg/m^2^第1天，每21天重复; EP组接受足叶乙甙80 mg/m^2^第1-5天，顺铂25 mg/m^2^第1-3天，每21天重复。每个周期复查血常规、肝肾功，记录化疗相关毒副反应，每两个周期行增强CT、肿瘤标志物、酌情行骨扫描及头颅MRI检查疗效评价。在治疗过程中如发生骨髓抑制，立即予粒细胞集落刺激因子治疗，至血象恢复正常后再行下一周期化疗，化疗期间予昂丹司琼预防性止吐。若疾病进展或出现严重毒副反应时停止研究治疗。

### 1.4 疗效及毒副反应评价

疗效评价按照实体瘤RECIST标准，分为完全缓解（complete response, CR）、部分缓解（partial response, PR）、疾病稳定（stable disease, SD）、疾病进展（progressive disease, PD）; 缓解率（response rate, RR）为达到CR、PR者百分比，疾病控制率（disease control rate, DCR）为达到CR、PR、SD者百分比。毒副反应根据WHO毒性反应评定标准分为0度-Ⅳ度。

### 1.5 随访与统计学方法

患者化疗期间每两周到医院或通过电话随访，化疗结束后每月进行门诊或电话随访，直至患者死亡或随访截止时间。随访时间为2006年4月-2012年1月，绝大部分患者在2年内死亡，大部分患者的随访时间即为其生存时间，EO组、EP组中位随访时间分别为11.2个月和9.1个月。随访过程中通过专人负责、预留多个联系方式、患者教育等方式尽量避免其它因素影响，随访内容主要为患者生存情况、疾病进展情况及治疗情况。主要研究终点是总生存时间（overall survival, OS）、无疾病进展生存期（progression free survival, PFS）和毒副反应。

### 1.6 统计学分析

统计分析使用SPSS 13.0统计软件，基线情况和疾病控制率、缓解率的比较采用χ^2^检验，OS及PFS采用*Kaplan Meier*法分析，采用*Log-rank*检验分析差异，*P* < 0.05为差异有统计学意义。

## 2 结果

### 2.1 疗效评价

EO组1例患者因急性阑尾炎手术仅完成1个周期化疗，而EP组1例患者因出现有症状脑转移仅完成1个周期化疗。69例患者至少完成2个周期化疗。EO组34例患者共完成130个周期EO方案化疗，平均每位患者完成3.82个周期; EP组35例患者共完成111个周期EP方案化疗，平均每位患者完成3.17个周期。EO组患者完成治疗情况优于EP组患者。

EO组34例患者中CR 3例，PR 16例，SD 9例，PD 6例，疾病控制率为82.4%（28/34），缓解率为55.9%（19/34），中位PFS为5.5个月，中位OS为10.5个月。EP组35例患者中CR 2例，PR 17例，SD 8例，PD 8例，疾病控制率为77.1%（27/35），缓解率为54.3%（19/35），中位PFS为4.7个月，中位OS为9.1个月。EO组与EP组相比，缓解率（55.9% *vs* 54.3%, *P*=0.894）、疾病控制率（82.4% *vs* 77.1%, *P*=0.591）、中位PFS（5.5个月*vs* 4.7个月，*P*=0.638，图 1A）和中位OS（10.5个月*vs* 9.1个月，*P*=0.862，图 1B）差异均无统计学意义（表 2）。

**1:**
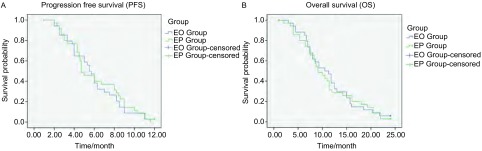
EO组和EP组的无疾病进展期（A）和总生存期（B）的比较 The comparison of progression free survival (A) and overall survival (B) in EO and EP groups

**2:** 足叶乙甙联合奥沙利铂和足叶乙甙联合顺铂的疗效评价（*n*=69） Efficacy evaluation of etoposide plus oxaliplatin (EO) and etoposide plus cisplatin (EP) (*n*=69)

Efficacy	EO group (*n*=34)	EP group (*n*=35)	*P*
CR	3	2	-
PR	16	17	-
SD	9	8	-
PD	6	8	-
RR	55.9%	54.3%	0.894
DCR	82.4%	77.1%	0.591
PFS (month)	5.5	4.7	0.638
OS (month)	10.5	9.1	0.862
CR: completely response; PR: partial response; SD: stable disease; PD: progressive disease; RR: response rate; DCR: disease control rate; PFS:progression free survival; OS: overall survival.			

### 2.2 毒性反应

71例患者均可评价毒性反应。两组最常见的毒性反应为骨髓抑制，表现为白细胞降低、血小板减少、贫血，多为Ⅰ级-Ⅱ级。EO组Ⅰ级-Ⅱ级神经毒性的发生率较EP组高（74.3% *vs* 11.1%, *P* < 0.001），但并未发生Ⅲ级以上神经毒性，不对患者的后续治疗及生活质量产生严重影响。EO组的恶心呕吐发生率明显低于EP组（65.7% *vs* 97.2%, *P*=0.001），且未发生Ⅲ级-Ⅳ级恶心呕吐（0 *vs* 16.7%, *P*=0.036）。EP组3例（8.3%）患者出现肾功能损害。本研究中两组患者均未出现治疗相关性死亡。相关的毒性反应发生情况见表 3。

**3:** 足叶乙甙联合奥沙利铂和足叶乙甙联合顺铂的毒性反应（*n*=71) Toxicities of etoposide plus oxaliplatin (EO) and etoposide plus cisplatin (EP) regimens (*n*=71)

Toxicity	EO group (*n*=35)					EP group (*n*=36)			
	GradeⅠ	GradeⅡ	Grade Ⅲ	Grade Ⅳ		GradeⅠ	GradeⅡ	Grade Ⅲ	Grade Ⅳ
Leukopenia	9 (25.7%)	13 (37.1%)	8 (22.9%)	4 (11.1%)		9 (25%)	13 (36.1%)	8 (22.2%)	6 (16.7%)
Thrombocytopenia	14 (40.0%)	8 (22.9%)	5 (14.3%)	2 (5.7%)		11 (30.6%)	6 (16.7%)	4 (11.1%)	2 (5.6%)
Anemia	11 (31.4%)	7 (20%)	1 (2.9%)	0		16 (44.4%)	7 (19.4%)	3 (8.3%)	0
Nausea/vomiting	15 (42.9%)	8 (22.9%)	0	0		20 (55.6%)	9 (26%)	4 (11.1%)	2 (5.6%)
Impaired liver function	4 (11.4%)	0	0	0		2 (5.6%)	1 (2.8%)	0	0
Impaired renal function	0	0	0	0		2 (5.6%)	0	1 (2.8%)	0
Neurocytoxicity	17 (48.6%)	9 (25.7%)	0	0		3 (8.3%)	1 (2.8%)	0	0
Fatigue	2 (5.7%)	0	0	0		2 (5.6%)	1 (2.8%)	1 (2.8%)	0

## 3 讨论

随着年龄的增长，肺癌的发生率逐渐升高，约一半的肺癌患者在确诊时已超过70岁，而老年肺癌患者由于伴有慢性疾病、器官功能减退等，对化疗的耐受性较差。SCLC恶性程度高，大多数患者在确诊时已出现远处转移，而无法得到根治，手术在其综合治疗中的价值非常有限，但SCLC对化疗较敏感，因此化疗对SCLC患者缓解症状、提高生活质量、延长生存期的作用至关重要。近年来针对非小细胞肺癌出现了靶向治疗等新的手段，产生了较好的效果，然而对于广泛期SCLC，目前依托泊苷联合顺铂仍然是最常用的一线化疗方案，但顺铂容易导致严重的恶心呕吐反应，影响患者的生活质量及化疗的进行。以卡铂或顺铂为基础的双药方案一线治疗SCLC的疗效相当^[5, 6]^，目前临床常用卡铂代替顺铂，以降低发生呕吐、神经毒性及肾毒性的风险，但由于骨髓抑制是卡铂最常见的毒副作用，其用于老年SCLC中所致的骨髓抑制亦常影响化疗的进行，并且可能威胁患者的生命安全。奥沙利铂是第三代铂类抗肿瘤药物，它与DNA的结合速度为顺铂的10倍以上，具有更强的细胞毒作用^[7, 8]^。目前奥沙利铂应用于结直肠癌等实体肿瘤中疗效确切且毒副反应发生率低，文献^[9]^报道奥沙利铂单药（130 mg/m^2^，每21天）应用于结直肠癌患者时3级-4级胃肠道毒性、神经系统毒性、血液学毒性发生率分别为0.1%、0.06%和0.13%。此外，在与一些化疗药物联合应用于非小细胞肺癌时，奥沙利铂也显示出了更低的消化道反应、血液学毒性和肝肾功能损害，而疗效并不劣于顺铂^[10, 11]^。另据文献^[12]^报道，奥沙利铂作为铂类药物联合依托泊苷应用于初治的广泛期SCLC患者的有效率为61.1%，证明了其应用于SCLC的可行性。

本研究探索了足叶乙甙联合奥沙利铂与顺铂一线治疗老年广泛期SCLC患者的疗效和不良反应。结果显示，试验组与对照组的缓解率差异无统计学意义（55.9% *vs* 54.3%, *P*=0.894），但对照组略低于国外文献^[13]^既往报道的60%-70%，可能与本研究样本量较小，造成了选择性偏倚有关。本研究中EO组与EP组的缓解率、控制率、PFS、OS差异均无统计学意义，但EO组似乎稍优于EP组，本研究例数有限，尚需大样本前瞻性临床试验进一步研究。在毒副反应方面，EP组恶心呕吐发生率明显高于EO组（97.2% *vs* 65.7%, *P*=0.001），且Ⅲ级-Ⅳ级恶心呕吐发生率亦较高（*P*=0.036）。然而老年人胃肠功能不佳，胃液分泌减少和粘液-碳酸氢盐屏障的破坏常导致慢性胃炎或消化系统溃疡，另外胰腺外分泌及胆盐的减少、结肠排空减慢等均对老年人的消化功能造成不良影响^[14]^。EP方案化疗导致的严重恶心呕吐反应加重了患者的胃肠道负担，使身体机能恢复减慢，降低对化疗的耐受，可导致化疗延期甚至中断，这对于进展速度较快的SCLC患者是极度不利的，同时也成为影响患者生存质量的主要因素。本研究中，EP组平均每位患者完成3.17个周期，EO组平均每位患者完成3.82个周期，表明EO组完成化疗情况优于EP组。此外，研究者观察到EP组有3例（8.3%）患者出现肾功能损害，虽经治疗后均好转，但较未发生肾功能损害事件的EO组相比安全性显然不及后者。值得一提的是，EO组的神经毒性发生率明显高于EP组（74.3% *vs* 11.1%, *P* < 0.001），但均为Ⅰ级-Ⅱ级，对后续治疗及生存质量无明显影响。奥沙利铂相关的急性神经病变，以感觉障碍和感觉异常为主，发生多较短暂，可自行恢复; 其相关的慢性神经病变多为积累毒性所致。在关于结直肠癌患者应用奥沙利铂联合方案的研究^[15]^报道中显示，第1个周期化疗后患者感觉障碍和感觉异常持续的中位时间分别仅为5天和7天，但在化疗12个周期后增加到约21天，3个周期和6个周期后需要奥沙利铂减量的患者分别约2.7%和20%。对于广泛期SCLC患者的标准化疗推荐4个-6个周期，本研究中EO组患者平均化疗3.82个周期，绝大多数患者对奥沙利铂所致神经毒性可耐受，但需要提出的是，多周期应用的患者仍应考虑其积累毒性，应根据患者情况调整化疗药物。

综上所述，EO方案用于广泛期SCLC患者与EP方案疗效相当，但EO组恶心呕吐等对患者治疗及生存质量影响较大的消化道不良反应发生率低于EP组，老年患者更易耐受，值得进一步进行大样本、多中心的临床研究。
